# An optimized EfficientNetB7 deep learning model for lung cancer tissue pathology image recognition

**DOI:** 10.3389/fonc.2026.1862407

**Published:** 2026-09-22

**Authors:** Xiongwen He, Lili Fu, Baicheng Xu, Jingru Luo, Junnv Xu, Shu Lin, Mingfa Wang, Wenjun Tang

**Affiliations:** 1 Department of Medical Oncology, The Second Affiliated Hospital of Hainan Medical University, Haikou, Hainan, China; 2 Department of Pathology, The Second Affiliated Hospital of Hainan Medical University, Haikou, Hainan, China

**Keywords:** convolutional neural networks, deep learning, lung cancer, pathological images, recognition

## Abstract

**Background:**

Lung cancer is the leading cause of cancer deaths worldwide. This study constructed and optimized a deep learning model to recognize lung cancer pathological images for intelligent classification.

**Methods:**

The LC25000 dataset was adopted for model training, and the LungHist700 dataset served as independent validation, with validation F1 score as the primary evaluation metric. Nine classic convolutional neural networks (DenseNet201, EfficientNetB7, EfficientNetV2S, InceptionResNetV2, MobileNetV3Large, NASNetLarge, ResNet50V2, VGG19, and Xception) were first benchmarked. Afterwards, three ensemble strategies including Equal-Weighted Averaging, Performance-Weighted Averaging and XGBoost were tested. Meanwhile, hyperparameter tuning was implemented on the optimal single model.

**Result:**

All nine baseline models obtained good performance on training and internal test datasets. Among individual models, EfficientNetB7 performed best with an F1 score of 0.891 and AUC of 0.749. Ensemble methods only reached a maximum F1 of 0.889, showing no obvious advantage over the best single model. By contrast, the hyperparameter-optimized EfficientNetB7 achieved remarkable generalization improvement, with accuracy 0.873, precision 0.864, recall 0.999, F1 score0.926 and AUC 0.871.

**Conclusion:**

The optimized model effectively recognizes lung cancer pathological images and supplies technical support for intelligent diagnosis. Multi-center large-sample clinical validation is required for future clinical transformation.

## 1 Introduction

Lung cancer is one of the most threatening malignant tumors to human health worldwide. The latest global cancer statistics show that lung cancer ranks among the top in both new cases and deaths from all malignant tumors, placing a heavy burden on global public health systems (1, 2). Early diagnosis and accurate subtyping of lung cancer are crucial for improving patient prognosis and reducing mortality (3), while histopathological biopsy is the gold standard for lung cancer diagnosis and the basis for subsequent treatment plans (4). Traditional pathological diagnosis relies on the professional experience and judgment of pathologists, which may have limitations. First, there is a shortage of pathologists globally, especially in primary healthcare institutions, where the scarcity of pathological resources may limit the ability to diagnose lung cancer early (5). Second, the examination of pathological slides is time-consuming and labor-intensive, leading to potential errors (6, 7). Furthermore, pathologists with different levels of experience may produce varying diagnostic results for the same pathological slide, requiring improvements in diagnostic consistency (8). With the rapid development of digital pathology technology and the gradual popularization of whole-slide imaging (9), the digital storage and transmission of pathological slides have become commonplace, providing a foundation for AI-based automated pathological diagnosis (10, 11).

In recent years, Vision Transformers and large-scale pathology foundation models like UNI, Virchow, CONCH, and TransVG++ have shown effective feature extraction and transfer capabilities in pathology image classification tasks (12, 13). By self-supervised pretraining on a large number of pathology images, these models can learn more general representations of tissue morphology. However, deploying these models usually requires more computing resources and memory, and their performance in small-sample fine-tuning scenarios still needs more empirical validation (14). Deep learning, as a branch of artificial intelligence, has demonstrated high performance in computer vision tasks and has been widely applied in various scenarios of medical image analysis (15, 16). In the field of pathological image analysis, deep learning technology can automate feature extraction, classification, and recognition of cancer tissue pathological images, reducing the workload of pathologists and improving diagnostic efficiency and consistency (17). Previous studies have applied deep learning technology to the classification task of lung cancer pathological images, confirming the feasibility and effectiveness of convolutional neural networks (CNNs) in lung cancer pathological image recognition (18–20).

Despite the progress made in existing research, several issues remain to be addressed. Some studies only divide the training and testing datasets within a single dataset, failing to validate the models using independent external datasets (21, 22). This leads to an overestimation of the models’ generalization performance, potentially failing to reflect their effectiveness in real-world clinical scenarios (23, 24). Furthermore, existing research often employs a single CNNs architecture for model construction, lacking benchmark testing for lung cancer pathology image classification tasks. Model selection lacks detailed justification, making it difficult to determine the optimal network architecture (19, 23). Moreover, while some studies have used ensemble strategies to improve model performance, most have not compared the differences between different ensemble strategies, failing to clarify whether ensemble models significantly outperform the optimal single model, thus lacking validation of the application value of ensemble strategies (25).

Based on the aforementioned research status and challenges, this study aims to construct and optimize a deep learning-based lung cancer tissue pathology image recognition model. First, two completely independent public datasets are used, one for model training and the other for independent external validation. The F1 score on the validation dataset is used as the selection metric to benchmark nine classic CNNs architectures, identifying the baseline model with the best generalization performance. Subsequently, three different ensemble strategies are employed to construct an ensemble model based on the output of the benchmark model, evaluating the application value of ensemble strategies in this work. Finally, hyperparameter optimization is performed on the optimal single model to explore its performance potential, ultimately constructing the final lung cancer tissue pathology image recognition model.

This research has both theoretical and practical value. Through benchmark testing, it clarifies the differences in generalization performance of different CNNs architectures in lung cancer pathological image binary classification tasks, providing a benchmark reference for model selection in this field. Simultaneously, by comparing the performance of different ensemble strategies, it clarifies their application value and limitations, providing important reference for related research. In practice, the optimized deep learning model constructed in this study may demonstrate generalization performance on independent external validation datasets, achieving accurate identification of lung cancer tissue pathological images. This provides technical support for the development of automated lung cancer pathological diagnostic tools, improves the efficiency and consistency of lung cancer pathological diagnosis, and promotes the enhancement of the ability of deep learning models to diagnose early lung cancer.

## 2 Materials and methods

### 2.1 Dataset and image preprocessing

This study used two publicly available lung cancer histopathological image datasets, LC25000 and LungHist700, for experiments. LC25000 contains hematoxylin-eosin stained images of normal lung tissue and lung cancer tissue (26). This dataset employs augmentation strategies such as flipping and rotation to increase the sample size. The analysis in this work excludes normal colon tissue and colon cancer samples from the dataset. Furthermore, different histological subtypes of lung cancer are merged.

All samples were selected and stratified randomly into training and testing dataset at an 8:2 ratio to ensure balanced class distribution. This dataset was used solely for model training and internal testing. The total number of samples in this dataset is 15,000, with 10,000 in the disease group and 5,000 in the control group. In training, we sample disease cases to balance the samples between the two groups. LungHist700 served as an independent external validation dataset (27), not involved in training or parameter tuning, and was used to evaluate the model’s generalization ability. The total number of samples in this dataset is 691, with 540 in the disease group and 151 in the control group.

All images were preprocessed using standardization. They were uniformly scaled to 224×224 pixels to fit the network input; pixel normalization and standardization were performed based on the training dataset’s mean and standard deviation to improve training stability. Data augmentation strategies such as random flipping, rotation, and brightness/contrast adjustment were employed on the training dataset to increase data diversity and suppress overfitting. This study used Pillow 12.3.0 and torchvision 0.28.0 for image processing, and scikit-learn 1.4.0 for dataset partitioning.

### 2.2 Construction of the benchmark deep learning models

This study selects nine classic CNN architectures—DenseNet201, EfficientNetB7, EfficientNetV2S, InceptionResNetV2, MobileNetV3Large, NASNetLarge, ResNet50V2, VGG19, and Xception—to construct benchmark models, covering different network depths, widths, and feature extraction modes, providing a reference for performance comparison. All models employ transfer learning strategies, loading ImageNet pre-trained weights to initialize parameters, reusing general image feature extraction capabilities, reducing data dependence, and improving training efficiency and generalization.

This study used networks pre-trained on ImageNet, with the final binary classification fully connected layer randomly initialized. The entire training process involved full-parameter fine-tuning, meaning all layers participated in gradient updates to adapt to pathological image features. Random horizontal and vertical flips were applied to the training dataset with a probability of 0.5, random rotations within ±25°, and brightness and contrast adjustments in the 0.8-1.2 range. The validation and test datasets only underwent size normalization and pixel standardization, without any random transformations. The number of training iterations and learning rate were fixed.

The model training was configured with an output dimension of 1 to adapt to binary classification tasks, and the output layer used the sigmoid activation function to generate predicted probabilities. All models were trained with the same hyperparameters: batch size 32, Adam optimizer, initial learning rate 1e-4, cross-entropy loss function, and 30 training epochs. The entire model implementation was based on PyTorch 2.2.0, and the pre-trained weights were derived from torchvision models and timm 0.9.16 (https://github.com/huggingface/pytorch-image-models).

### 2.3 Construction and training of ensemble models

This study also employs three ensemble strategies Equal-Weighted Averaging, Performance-Weighted Averaging, and eXtreme Gradient Boosting (XGBoost)—to quantify the performance improvement of ensemble models in lung cancer identification. A basic CNN performs preliminary feature classification of pathological images, and the raw output scores (Raw Score) of each model before sigmoid activation are extracted as feature vectors and input into the ensemble learning model for final classification prediction. Compared to probability values compressed to the 0–1 range, unbounded raw scores fully preserve prediction confidence information, avoiding the discrimination loss caused by prediction probabilities. Their distribution characteristics are more compatible with the logic of ensemble models, enhancing the ensemble model’s ability to capture differences in the outputs of multiple models. Equal-Weighted Averaging uses an equal weighted average of the prediction scores of the nine models. Performance-Weighted Averaging uses the Train or Test dataset F1 score as a weight for a weighted average, resulting in a higher contribution from superior models (In ensemble models, the weights are preferably based on the results from the Train dataset; if the weights are consistent in the Train dataset, then the Test dataset is used).

XGBoost constructs a binary classification model using raw scores as features. The nine base CNNs’ raw scores on the training dataset were then extracted as a 9-dimensional feature vector to train the XGBoost meta-classifier. For evaluation, raw scores generated by the trained base CNNs on the internal test dataset and the validation dataset were fed into the trained XGBoost model to produce final predictions. Neither the internal test dataset nor LungHist700 was used to train the XGBoost meta-classifier. This study used Spearman rank correlation analysis to evaluate the performance correlations between individual deep learning models and performed statistical significance tests on the resulting correlation coefficients. The study set a threshold of an absolute correlation coefficient > 0.3 and a significance test P < 0.05 as the criteria for considering a substantial correlation. The ensemble models is implemented using xgboost 2.0.3 and NumPy 1.26.3.

### 2.4 Hyperparameter optimization

To verify the model’s generalization ability, this study inputs a validation dataset (not used in training) into the optimal model and uses the output evaluation metrics as the basis for generalization performance. Five metrics are used to quantify classification performance: accuracy, precision, recall, F1 score, and area under the curve (AUC) of receiver operating characteristic (ROC). The F1 score is the core metric for model selection and hyperparameter optimization, while AUC assesses the model’s discriminative ability. If the difference in F1 scores between models is within 0.01, the highest AUC is used as a secondary criterion for evaluation. This study performs 3000 hyperparameter optimization iterations on the best-performing single model, covering 7 types of parameters: learning rate, batch size, dropout rate, loss function, weight decay, training epochs, and optimizer. The optimal combination is then selected for retraining to obtain the final model.

The hyperparameter random search space contains 7 types of hyperparameters. (1) The learning rate (lr) uses a logarithmic floating-point search method, with a value range of 1e-5 to 1e-3, divided into 10 segments; (2) The batch size (batch_size) is used for classifying candidate value search, with values including 8, 16, 32, and 64; (3) The optimizer is used for classifying candidate value search, with candidate types covering three optimizers: adam, sgd, and adamW; (4) The weight decay (weight_decay) uses a logarithmic floating-point search, with a value range of 1e-5 to 1e-3, used to control model overfitting through L2 regularization; (5) The dropout rate (dropout_rate) uses a linear floating-point search, with a value range from 0.0 to 0.5, used to adjust the regularization strength of the model; (6) The loss function (loss_fn) is used for classifying candidate value search, with optional types including binary cross-entropy loss (bce), loss function with logits (binary cross-entropy loss, bce_with_logits), and focal loss; (7) The number of training epochs (num_epochs) is an integer search, ranging from 10 to 30 training epochs, which balances the sufficiency and efficiency of model training. During the model optimization phase, training only happens on the train dataset, while independent evaluation of the model is done on the validation dataset. The optimal hyperparameter configuration was selected according to the same principle applied to the single models, with the validation F1 score as the primary criterion and the AUC serving as the secondary criterion when the F1 difference was within 0.01.

### 2.5 Model validation, evaluation indicators and statistical analysis

The raw scores of each basic model, together with the predicted probabilities of ensemble models and the optimized model, were extracted to characterize the score distributions between normal and lung cancer samples. Accuracy, precision, recall, F1 score, AUC, and their 95% confidence intervals (CI) were also calculated. All calculations and statistical analyses were performed using scikit-learn and SciPy 1.12.0. The DeLong test was applied to compare paired ROC AUCs, while the McNemar test with continuity correction was performed on the 2x2 contingency table of correct/incorrect classifications. Brier score was computed as the mean squared difference between the predicted probabilities and the binary ground-truth labels, and expected calibration error (ECE) was calculated by partitioning samples into equal-width confidence bins and taking the sample-weighted average of the absolute difference between the in-bin average accuracy and in-bin average confidence. Unless otherwise specified, the significance threshold for statistical tests is set at p < 0.05.

All experiments were developed using Python 3.12.1, with Graphics Processing Unit (GPU) acceleration relying on CUDA 12.6 and cuDNN 8.9.7. The hardware environment consisted of an NVIDIA RTX 3090 GPU, an Intel i9–14900 CPU, and Windows 10 as the operating system. Image processing was performed using Pillow, data processing used Pandas 2.2.0, and visualization was implemented using Matplotlib 3.8.2.

## 3 Results

### 3.1 Performance of single models

The overall process and typical case images of this study are shown in **Figures 1A, B**, respectively. This study first benchmarked nine classic CNN models to evaluate the performance of different architectures in lung cancer pathological image classification. The results showed that all nine benchmark models achieved high fit performance in terms of accuracy, precision, recall, F1 score, and AUC on both the training and test datasets, with no significant underfitting issues during both training and internal testing (**Figures 2A–I**; **Supplementary Table A**). ROC analysis showed that all models had an AUC of 1.000 on both the train and test datasets, with 95% CI of [1.000, 1.000], further confirming the stable performance of the models during training and internal testing (**Figures 3A–I**). In the evaluation on the independent external validation dataset, the generalization performance of each model varied. The EfficientNetB7 model achieved high validation dataset F1 score (0.891) and AUC (0.749) among all benchmark models (**Figures 2B**, **3B**). The validation dataset F1 scores of DenseNet201, EfficientNetV2S, and InceptionResNetV2 models were 0.812, 0.893, and 0.880, respectively, showing high performance. The VGG19 model had the lowest validation dataset F1 score, only 0.494, indicating the worst generalization performance (**Figures 2H**, **3H**). The confusion matrix for the single model can be seen in **Supplementary Figure 1**. These results confirm that different CNN architectures exhibit varying generalization performance in lung cancer pathological image recognition tasks, with the EfficientNetB7 model demonstrating the best basic generalization ability in this task.

**Figure 1:**
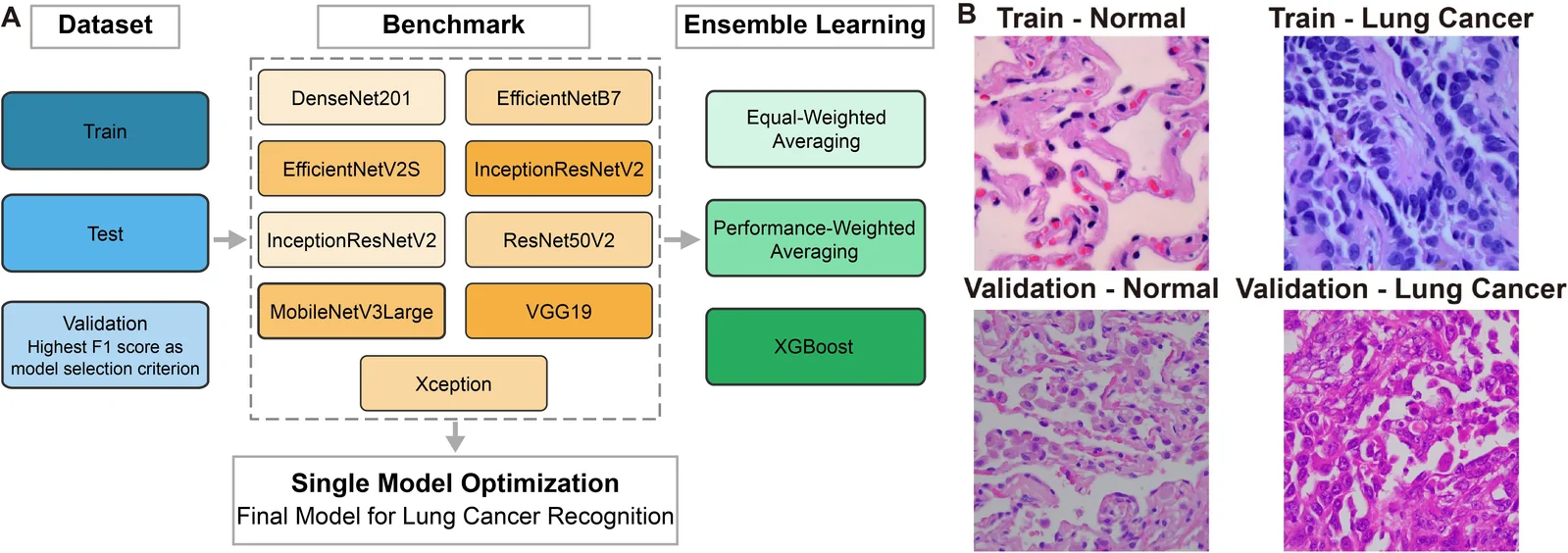
Research technique and examples of lung cancer tissue pathology image. **(A)** Overall technical approach to the study; **(B)** Examples of normal and lung cancer tissue pathology images from the training and validation datasets.

**Figure 2:**
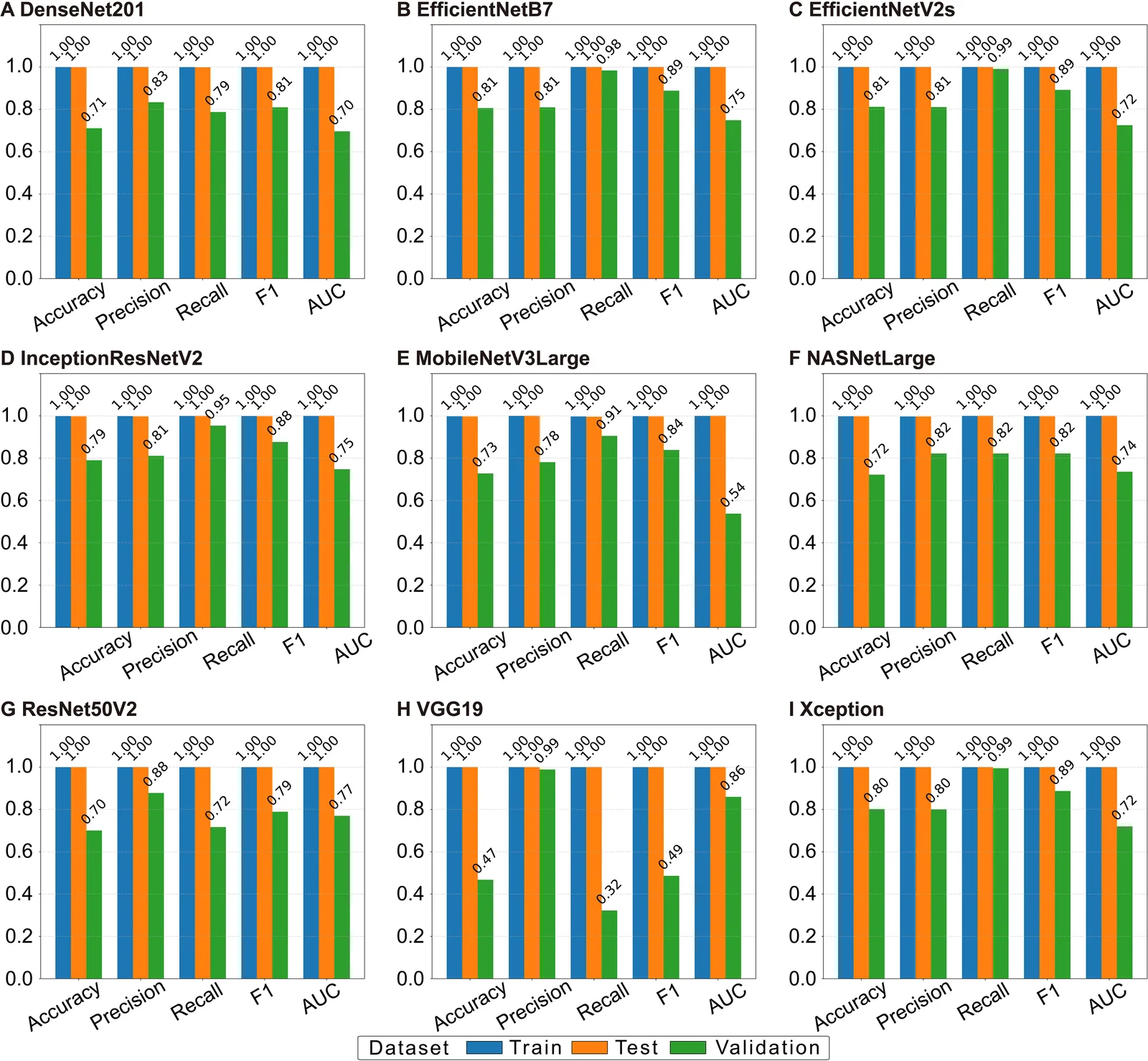
Performance of benchmark deep learning models. **(A)** DenseNet201; **(B)** EfficientNetB7; **(C)** EfficientNetV2S; **(D)** InceptionResNetV2; **(E)** MobileNetV3Large; **(F)** NASNetLarge; **(G)** ResNet50V2; **(H)** VGG19; **(I)** Xception. AUC, Area under the receiver operating characteristic (ROC) curve.

**Figure 3:**
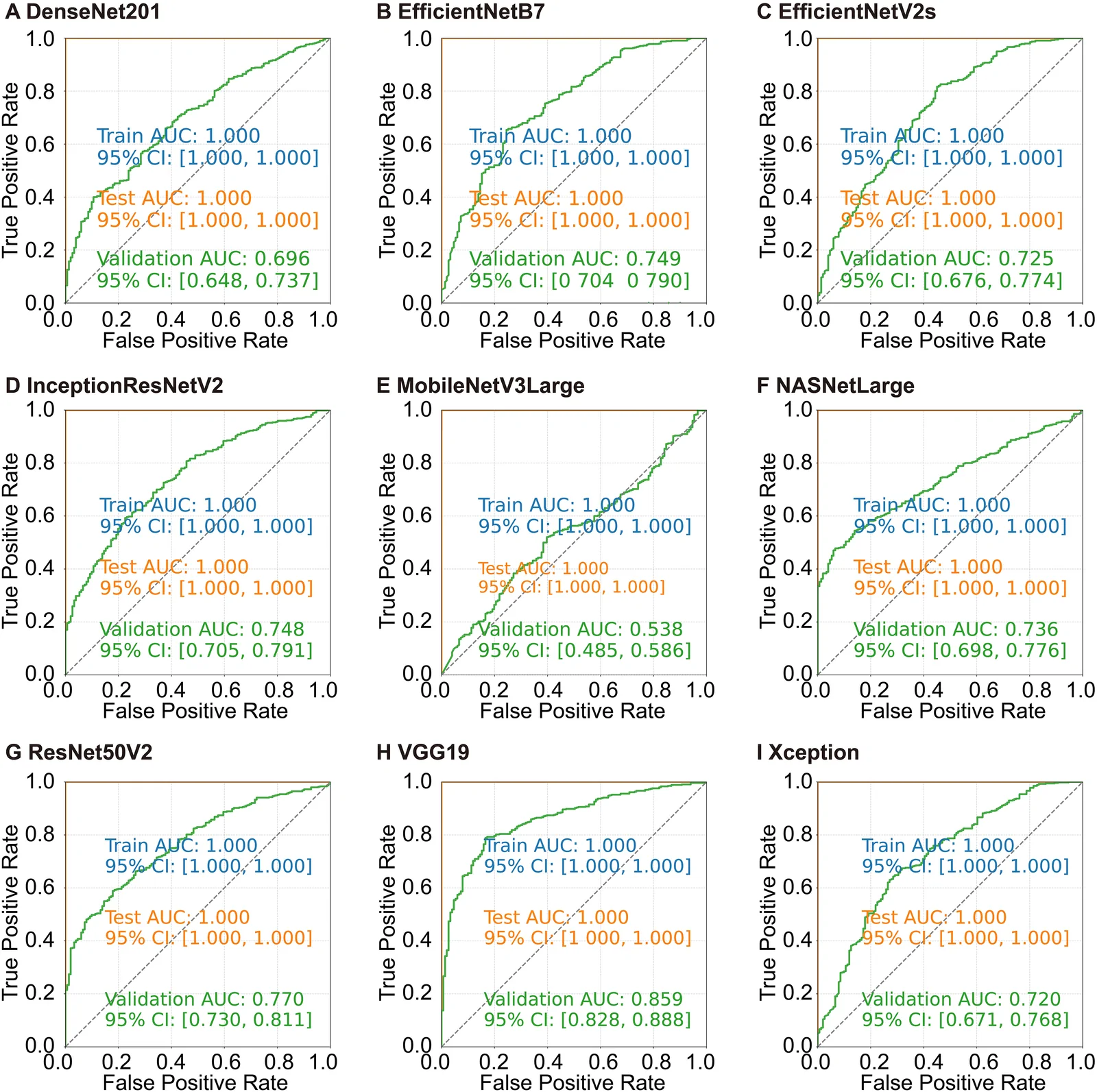
Receiver operating characteristic curve of the benchmark deep learning models. **(A)** DenseNet201; **(B)** EfficientNetB7; **(C)** EfficientNetV2S; **(D)** InceptionResNetV2; **(E)** MobileNetV3Large; **(F)** NASNetLarge; **(G)** ResNet50V2; **(H)** VGG19; **(I)** Xception. AUC, area under the receiver operating characteristic (ROC) curve; CI, confidence interval.

### 3.2 Performance of ensemble models

To explore the role of model ensemble in improving the generalization performance of lung cancer pathological image recognition, this study constructed ensemble models using three different strategies based on the output results of nine benchmark models, and compared the performance of different ensemble strategies. The results showed that the Equal-Weighted Averaging, Performance-Weighted Averaging, and XGBoost ensemble models all achieved near 1.000 on all evaluation metrics in both the train and test datasets, maintaining high fitting performance comparable to the benchmark models (**Figures 4A–C**; **Supplementary Table B**). ROC curve analysis showed that the AUC of all three ensemble models was 1.000 in both the train and test datasets, with a 95% CI of [1.000, 1.000], indicating no significant difference in performance compared to the benchmark models (**Figure 4D**). In the evaluation on the independent external validation dataset, the F1 scores of the Equal-Weighted Averaging, Performance-Weighted Averaging, and XGBoost models were 0.889, 0.887, and 0.883, respectively, and their AUCs were 0.803, 0.807, and 0.800, respectively. None of these exceeded the validation dataset F1 score of the best single model, EfficientNetB7, in the benchmark data test. All ensemble strategies did not bring significant improvement in generalization performance (**Figures 2**–**4**). A pairwise Spearman rank correlation analysis of 9 deep learning models on the train, test, and validation datasets shows that the models are substantially positively correlated in both the train and test datasets, with most of the pairwise comparisons having P values less than 0.001, indicating strong statistical significance. There is also a clear correlation among models on the validation dataset (**Supplementary Figure 2**). The confusion matrix of the ensemble model is shown in **Supplementary Figure 3**. The above results confirm that in the lung cancer pathology image recognition task of this study, the multi-model-based ensemble strategies failed to significantly improve the generalization performance of the model. The best single model outperformed the ensemble model. Therefore, future research will focus on the hyperparameter optimization of the best single model, EfficientNetB7, to further explore the model’s performance potential.

**Figure 4:**
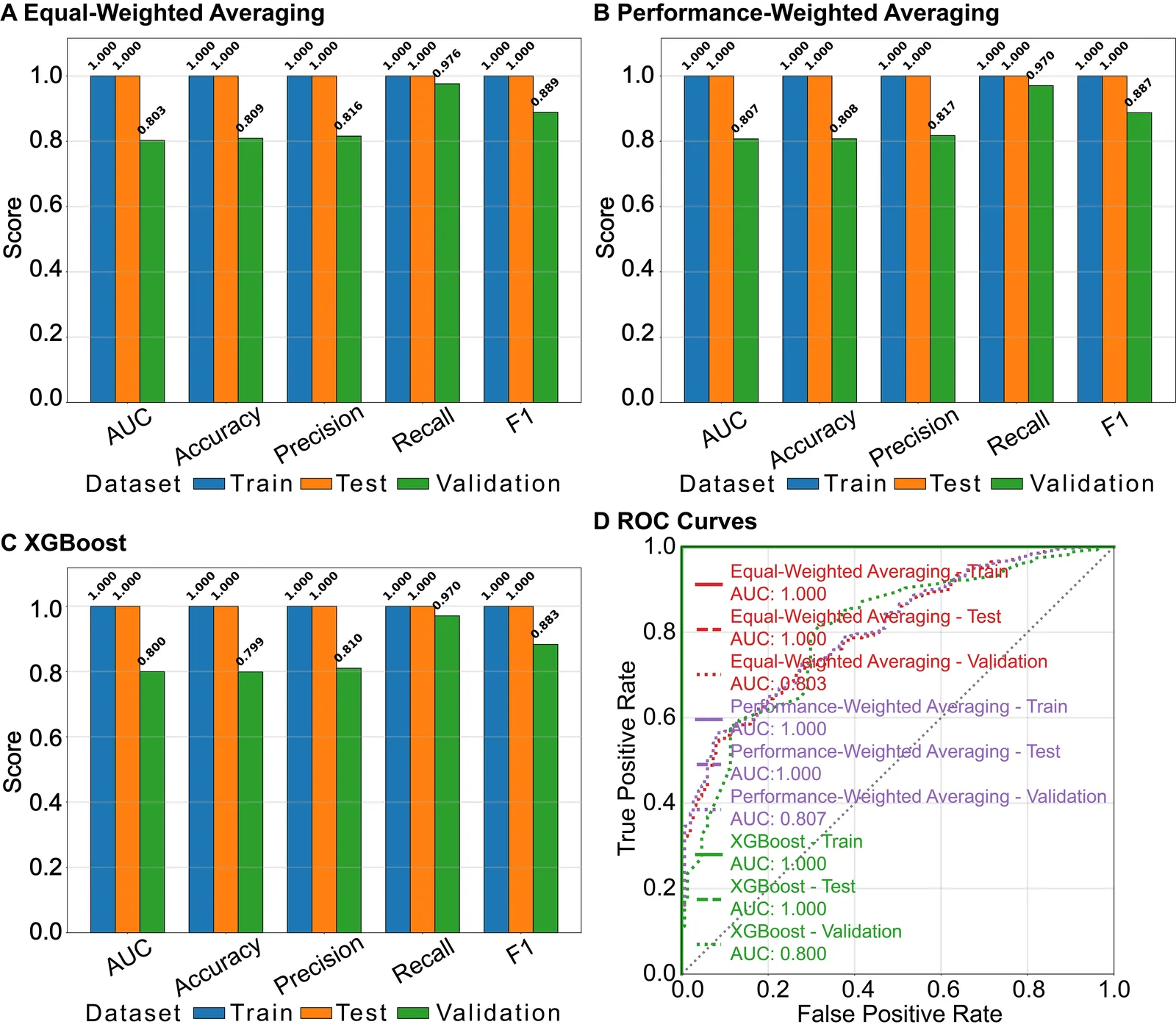
Performance of the ensemble learning model. **(A)** Classification performance of the Equal-Weighted Averaging model; **(B)** Classification performance of the Performance-Weighted Averaging model; **(C)** Classification performance of the XGBoost model; **(D)** Receiver operating characteristic (ROC) curves of the three ensemble learning models. XGBoost, Extreme Gradient Boosting; AUC, Area under the receiver operating characteristic; CI, confidence interval.

### 3.3 Performance of optimized single model

EfficientNetB7 delivered an F1 score of 0.891, essentially on par with EfficientNetV2S (0.893), and achieved the highest AUC 0.749 among all benchmark models. With F1 score taken as the primary evaluation metric and AUC as the supplementary criterion, EfficientNetB7 was selected as the model for subsequent hyperparameter optimization. To further improve the generalization performance of the EfficientNetB7 model, this study conducted optimization analysis on 7 core hyperparameters to clarify the impact of different hyperparameters on model performance and to discover the optimal parameter combination. The results showed that when the batch size was 16, the model’s validation dataset F1 score remained stable. Significant fluctuations in model performance occurred when the batch size increased to 32 or 64, and some fluctuations also occurred when the batch size was 8 (**Figure 5A**). When the dropout rate was between 0.00 and 0.26, the model’s validation dataset F1 score remained at a stable high level. Significant fluctuations in model performance occurred when the dropout rate exceeded 0.26 (**Figure 5B**). Regarding the loss function, models based on binary cross-entropy and binary cross-entropy with logits outperformed those based on Focal Loss. Focus loss caused fluctuations in model performance, with the F1 score dropping below 0.90 in multiple trials (**Figure 5C**). The model performance was most stable when the weight decay coefficient was in the range of 1e-5 to 5e-5. As the weight decay coefficient increased, the model performance decreased, with the F1 score dropping below 0.75 in some experiments (**Figure 5D**). When the number of training epochs was between 25 and 30, the model’s validation dataset F1 score remained at a stable high level. When the number of training epochs was less than 15, the model showed obvious underfitting and low performance (**Figure 5E**). In terms of optimizers, the AdamW optimizer had the best and most stable overall model performance, followed by the Adam optimizer. The Stochastic Gradient Descent (sgd)optimizer caused significant fluctuations and a decrease in model performance, with the F1 score dropping below 0.85 in multiple experiments (**Figure 5F**). The confusion matrix analysis of the optimized EfficientNetB7 can be seen in **Supplementary Figure 4**.

**Figure 5:**
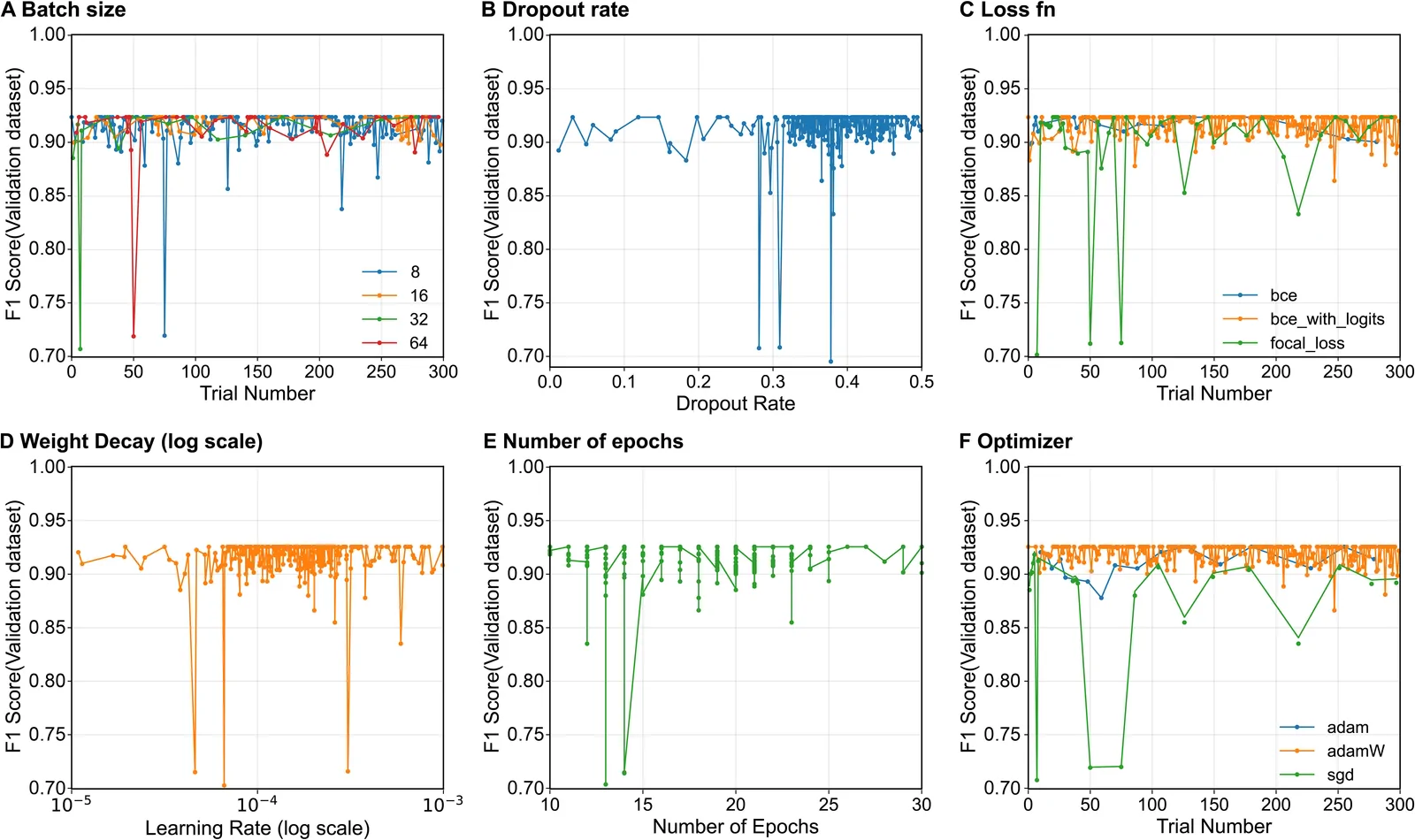
Hyperparameter optimization results of the EfficientNetB7 model. **(A)** The impact of batch size on model performance; **(B)** The impact of dropout rate on model performance; **(C)** The impact of loss function on model performance; **(D)** The impact of weight decay coefficient on model performance; **(E)** The impact of training epochs on model performance; **(F)** The impact of optimizer on model performance. bce, binary cross-entropy loss; bce_with_logits, binary cross-entropy loss with logits; focal_loss, focal loss; adam, adaptive moment estimator optimizer; adamW, adaptive moment estimator optimizer with weight decay correction; sgd, stochastic gradient descent optimizer.

The final model parameters were: batch size of 16, dropout rate of 0.3, using a binary cross-entropy optimization with logits and the AdamW optimizer, weight decay coefficient of 1e-4, and training epochs of 30. Based on the above hyperparameter optimization results, this study selected the hyperparameter combination with the highest validation dataset F1 score and retrained to obtain the optimized EfficientNetB7 model.

To verify the effectiveness of the hyperparameter optimization strategy, this study evaluated the performance of the optimized final model, clarifying its classification ability and generalization performance. The results showed that the optimized EfficientNetB7 model maintained high fitting performance with accuracy, precision, recall, F1 score, and AUC all at 1.000 on both the train and test datasets (**Figure 6A**). In the evaluation on the independent validation dataset, the optimized model achieved an accuracy of 0.873, precision of 0.864, recall of 0.999, an F1 score of0.926, and an AUC of 0.871. Compared to the base model before optimization, the validation dataset F1 score improved, and the AUC improved, demonstrating a significant improvement in generalization performance (**Figure 6A**; **Supplementary Table C**). ROC analysis results showed that the optimized model had an AUC of 1.000 on both the training and test datasets, with a 95% CI of [1.000, 1.000]; and an AUC of 0.871 on the independent external validation dataset, with a 95% CI of [0.796, 0.945], further confirming the stable generalization performance of the optimized model (**Figure 6B**). Furthermore, we use the DeLong test and McNemar test to compare the differences in AUC values and the number of correctly/incorrectly classified samples between the base EfficientNetB7 model and the final optimized model. The DeLong test yielded a Z-statistic of -5.508 (p < 0.001), indicating that the optimized model significantly outperformed the base EfficientNetB7 model. The McNemar test yielded a chi-square statistic of 6.223 (p = 0.013), confirming that the optimized EfficientNetB7 model had significantly higher classification accuracy than the base EfficientNetB7 model. These results demonstrate that the hyperparameter optimization strategy employed in this study can improve the generalization performance of the EfficientNetB7 model in lung cancer pathological image recognition. The optimized model can achieve accurate recognition of lung cancer tissue pathological images. We further calculated the Brier score and ECE for each model; the results showed that both metrics were low across all models, with the final optimized model achieving the lowest values for both Brier score and ECE on the validation dataset (**Supplementary Table D**).

**Figure 6:**
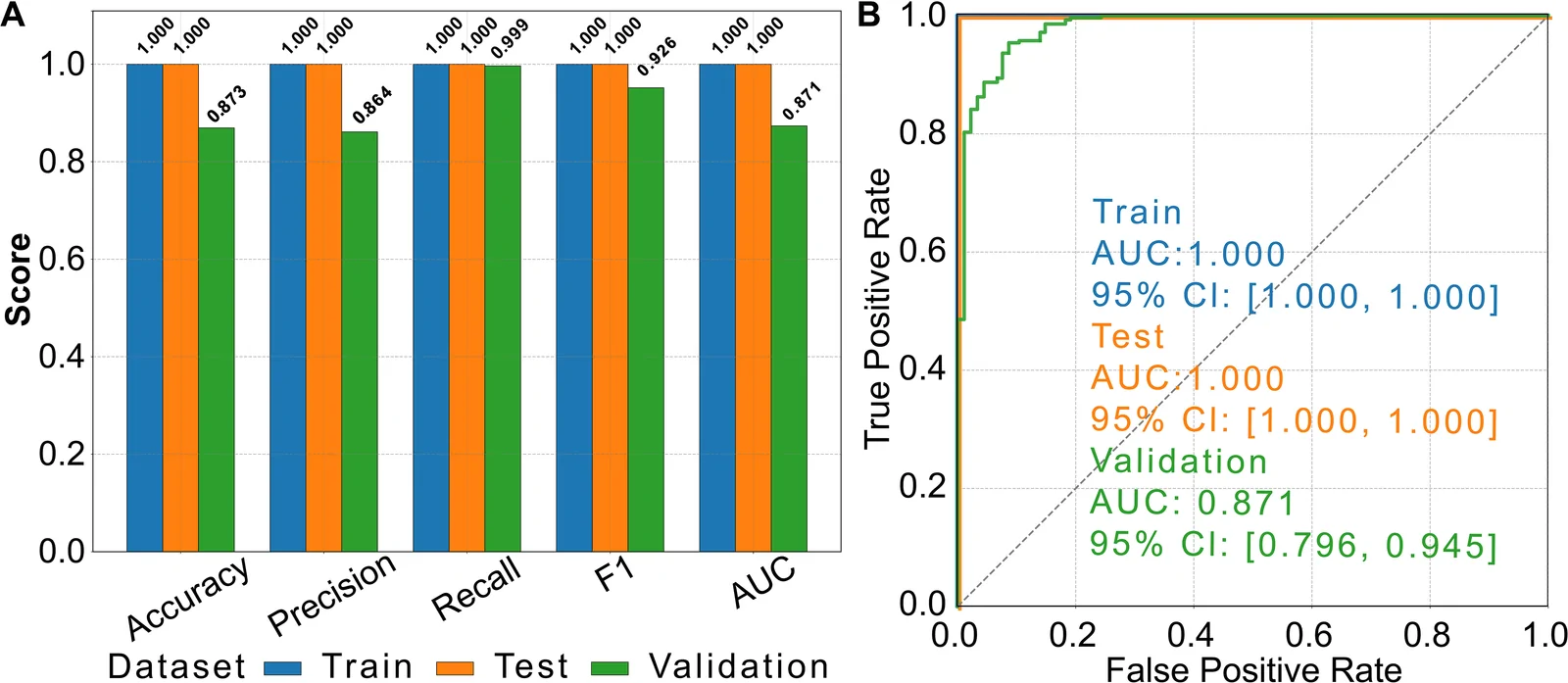
Performance of the optimized EfficientNetB7 model. **(A)** Classification performance of the optimized model; **(B)** Receiver operating characteristic (ROC) curve of the optimized model. AUC, Area under the receiver operating characteristic (ROC); CI, confidence interval.

## 4 Discussion

Lung cancer is the leading cause of cancer-related death worldwide, and histopathological diagnosis is the gold standard for lung cancer confirmation. However, traditional pathological diagnosis relies on the experience of pathologists, which may lead to subjectivity, inefficiency, and inconsistent results. Automated pathological image recognition technology has significant clinical needs and application value. This study benchmarked nine classic CNN models based on two completely independent lung cancer histopathological image datasets, compared the performance of three ensemble strategies, and optimized the hyperparameters of the optimal single model, EfficientNetB7, ultimately constructing a lung cancer pathological image recognition model with high generalization performance. The core findings of this study include: different CNNs architectures exhibit varying generalization performance in lung cancer pathological image recognition tasks, with EfficientNetB7 demonstrating the best basic generalization ability; ensemble strategies based on multiple models failed to improve the model’s generalization performance, with the optimal single model outperforming the ensemble model; hyperparameter optimization improved the generalization performance of the EfficientNetB7 model, achieving an F1 score of 0.926 and an AUC of 0.871 on the independent external validation dataset, demonstrating potential for clinical application.

The benchmark results of this study show that all nine CNNs models exhibit high classification performance on both the train and test datasets, but show differences in generalization performance on independent external validation dataset. This result is consistent with previous studies, which have shown that deep learning models often demonstrate high performance in partitioning tests within a single dataset, but their generalization performance drops significantly on independent external validation dataset (23, 24). The core reason for this phenomenon lies in the domain shift problem. Differences in pathological slide preparation procedures, staining conditions, scanning equipment, and parameters across different datasets lead to a shift in the data distribution of images. Features learned by the model on the training dataset are difficult to directly transfer to independent external datasets (28, 29). Besides, the augmentation strategies applied to the original dataset may have partially contributed to the very similar performance observed on the training and test datasets (26). However, due to project time and computing costs, our research only provides the benchmark performance of 9 algorithms with unified parameters. This study used two completely independent datasets for model training and generalization performance validation, simulating data differences in real-world clinical scenarios. Therefore, the model performance results obtained better reflect the actual performance of the model in real-world clinical applications.

Among the nine benchmark models, the EfficientNetB7 model exhibited the best generalization performance, a result closely related to the architectural design of the EfficientNet series models. The EfficientNet series models employ a composite scaling strategy, simultaneously optimizing the network’s depth, width, and input resolution. This improves the model’s feature extraction capabilities while controlling the number of parameters and computational complexity, reducing the risk of overfitting (30, 31). Previous research in medical image analysis has also confirmed that the EfficientNet series models demonstrate superior performance and generalization ability compared to other classic CNNs architectures in medical image classification tasks, consistent with the results of this study (22, 32). The VGG19 model, however, exhibited the worst generalization performance in this study. This is likely due to its relatively traditional architecture, employing only simple stacked convolutional layers, resulting in limited feature extraction capabilities. Furthermore, the fully connected layers contain a large number of parameters, making them prone to overfitting, leading to a significant drop in generalization performance, which aligns with previous related research findings (33).

This study compared the performance of three ensemble strategies. The results showed that Equal-Weighted Averaging, Performance-Weighted Averaging, and XGBoost failed to significantly improve the generalization performance of the models on the independent validation dataset. The F1 score of the ensemble model was even lower than that of the best single model, EfficientNetB7. This result differs from some previous studies, which showed that ensemble strategies can reduce the prediction variance of a single model and improve its classification performance and generalization ability by fusing features and predictions from multiple different models (34, 35). In this study, ensemble models did not bring significant performance improvements. The performance of the nine benchmark models on both the training and test datasets was high, suggesting that the prediction results between the models may lack sufficient complementarity, preventing ensemble models from achieving performance improvements by fusing multiple model results. Furthermore, some of the nine benchmark models showed poor generalization performance on the independent validation dataset. Integrating the results of these low-performance models into the ensemble model negatively impacted the overall result, offsetting the advantages of the high-performance model. This result also suggests that ensemble models do not always lead to performance improvement in medical image classification tasks. The reason the ensemble models aren’t performing well is probably because the results of the individual models are correlated, and all the models were pretrained using the same data and the same ImageNet initialization. These models are likely producing highly correlated errors, which limits the overall benefit.

Before model fusion, it is necessary to evaluate the performance and complementarity of the base models to avoid the negative impact of low-performance models on the ensemble results. In addition, targeted optimization for generalization performance is more effective than simple multi-model fusion in some cases.

This study optimized seven core hyperparameters of the EfficientNetB7 model. The results show that batch size, dropout rate, loss function, weight decay coefficient, training epochs, and optimizer all significantly impact the model’s generalization performance. The model retrained based on the optimal hyperparameter combination exhibited improved generalization performance. This result is consistent with previous research, which has shown that the choice of hyperparameters has a decisive influence on the training process and final performance of deep learning models. Hyperparameter optimization for specific tasks and datasets can effectively improve the model’s generalization performance and reduce the risk of overfitting (22, 30, 31). In this study, a batch size of 16 resulted in optimal model performance. The results indicate that excessively small batch sizes lead to unstable gradient updates, whereas excessively large batch sizes cause the optimization process to converge to sharp minima, thereby impairing the model’s generalization ability. This aligns with classic conclusions in deep learning optimization theory. An inappropriate Dropout rate can lead to a decline in model performance. A dropout rate > 0.26 generally leads to noticeable performance fluctuations. This study adopted a transfer learning strategy based on ImageNet pre-training, and the model’s basic feature extraction capabilities have already been trained. An excessively high dropout rate will destroy the effective features that the model has already learned, resulting in underfitting and a decline in performance. But in this study, the dropout in the optimized model is 0.3, which means some neurons are randomly deactivated during the forward pass of training. When used together with other parameters, this might prevent performance loss caused by relying too heavily on a single regularization method, and that’s why the highest F1 score was achieved on the validation dataset. In the end, the final parameter combination is the best solution from the multi-hyperparameter joint search for single-model parameter optimization in this study.

Regarding the loss function, the binary cross-entropy loss outperforms the Focal Loss. This is because the core function of Focal Loss is to address class imbalance in classification tasks by reducing the weight of easily classified samples and increasing the contribution of difficult-to-classify samples to the loss. However, the dataset used in this study has a balanced class distribution, and Focal Loss can actually lead to a deviation in the optimization direction of the model, affecting the overall performance of the model. As for the optimizer, the AdamW optimizer performs best. This is because the AdamW optimizer modifies the calculation method of weight decay based on the Adam optimizer, decoupling weight decay from gradient updates, effectively reducing the risk of overfitting and improving generalization performance. This is consistent with previous related research results study, through hyperparameter optimization, obtained the optimal hyperparameter combination for lung cancer pathological image recognition, improving the model’s generalization performance (22,^36^, ^37^). This demonstrates the importance of hyperparameter optimization in the construction of deep learning models for medical imaging and provides a reference for hyperparameter selection in related research. It is important to note that the patterns observed in the joint random search reflect correlational trends rather than causal effects established under controlled conditions. These results are intended to guide the selection range for hyperparameters and do not constitute definitive causal inference.

This study has several innovative aspects. First, it benchmarked nine classic CNN models for lung cancer pathological image recognition, using completely independent external datasets to evaluate their generalization performance and clarifying the performance differences between different architectures, providing a benchmark reference for model selection in this field. Second, it compared the performance of three ensemble strategies: Equal-Weighted Averaging, Performance-Weighted Averaging, and XGBoost, clarifying the application value and limitations of ensemble models in this task and providing important reference for the selection and application of ensemble strategies in related research. Third, it optimized 7 core hyperparameters for the optimal single model, constructing a lung cancer pathological image recognition model with high generalization performance, providing technical support for the development of automated pathological diagnosis tools for lung cancer. In short, this study used two datasets to complete a binary classification task on pathological images of normal lung tissue and lung cancer lesions. It evaluated the binary classification performance of nine deep learning algorithms, providing a basic reference for algorithm selection and performance benchmarking for this task.

This study also has certain limitations. First, the datasets used in this study are all publicly available datasets. Future research needs to further validate the model’s performance and generalization ability on real-world clinical datasets prepared across multiple centers, devices, and pathology departments. Second, this study only built the model for a binary classification task between normal lung tissue and lung cancer tissue, and did not cover more complex clinical tasks such as histological subtype classification, pathological grading, and TNM staging of lung cancer, nor did it perform data splitting and evaluation at the patient level or slice level. Future research could further expand the model’s application scenarios to meet more clinical needs. In addition, the original pathological images in the training dataset are augmented through operations such as rotation and flipping, which may cause the classification performance of the study results to decline in real scenarios. Augmentation based on limited original samples may introduce risks of data leakage and distribution bias, and will partially weaken the reliability of the model results.

In summary, this study is designed and positioned to conceptually solve the binary classification problem. Clarifying the binary classification performance of deep learning models is a necessary foundation for constructing multi-class differential diagnosis models. Limited by the augmentation strategy of the original dataset and sample imbalance, a cautious attitude should still be held toward the conclusions of this study, and its actual generalization ability remains to be further verified by real clinical samples.

Overall, the optimized EfficientNetB7 model constructed in this study can achieve accurate identification of lung cancer tissue pathology images, demonstrating good generalization performance in independent external validation datasets and showing potential for clinical application. It’s worth noting that the individual heterogeneity of lung cancer samples or the limitations of the algorithms currently used may still lead to a certain proportion of misclassified samples in the results of the optimized EfficientNetB7 model. Future research will focus on multi-center, large-sample clinical validation to expand the model’s application scenarios, improve its interpretability, promote the clinical translation of automated pathological diagnosis technology for lung cancer, and provide technical support for the early diagnosis and treatment of lung cancer.

## 5 Conclusion

Lung cancer is the leading cause of cancer-related morbidity and mortality worldwide. Histopathological diagnosis is the gold standard for lung cancer confirmation, and automated pathological image recognition technology can effectively improve the efficiency and consistency of pathological diagnosis, possessing significant clinical application value. This study aims to construct and optimize a deep learning-based lung cancer histopathological image recognition model, providing reference for automated pathological binary classification of lung cancer. This study constructed training and testing datasets based on the LC25000 dataset, using the LungHist700 dataset as an independent external validation dataset. Using the validation dastaset F1 score as the core metric, benchmark tests were conducted on nine CNNs models, and performance comparisons were performed on three ensemble strategies. This study optimized the hyperparameters of the EfficientNetB7 single model, which exhibited the best generalization performance in the benchmark tests, and constructed the final lung cancer histopathological image recognition model. The results show that EfficientNetB7 is the single model with the best generalization performance in the benchmark tests, and none of the three ensemble strategies improved generalization performance; the best single model outperformed the ensemble model. The hyperparameter-optimized EfficientNetB7 model achieved an F1 score of0.926 and an AUC of 0.871 on the independent validation dataset, demonstrating improved generalization performance compared to the base model before optimization. The optimized EfficientNetB7 model constructed in this study can achieve accurate identification of lung cancer tissue pathology images, providing technical support for the automation of lung cancer pathology diagnosis. This study clarified the high-performance model architecture and hyperparameter combinations for lung cancer pathology image recognition tasks, providing a benchmark reference for research in related fields. Future research will further conduct multi-center, large-sample clinical validation to expand the model’s application scenarios and promote its clinical translation.

## Data Availability

The original contributions presented in the study are included in the article/**Supplementary Material**. Further inquiries can be directed to the corresponding authors.
