# Supplementary material for: An optimized EfficientNetB7 deep learning model for lung cancer tissue pathology image recognition
Source: Front Oncol. 2026 Sep 22;16:1862407. doi: 10.3389/fonc.2026.1862407 (PMC13638370; doi:10.3389/fonc.2026.1862407)
Supplement: Supplementary file 1 [file DataSheet1.pdf]

An optimized EfficientNetB7 deep learning model for lung cancer tissue pathology image recognition

Xiongwen He[1#] • Lili Fu[1#] • Baicheng Xu[1] • Jingru Luo[1] • Junnv Xu[1] • Shu Lin[1]• Mingfa Wang[2*] • Wenjun Tang[1*]

[1]Department of Medical Oncology, The Second Affiliated Hospital of Hainan Medical University, Haikou 570311, Hainan Province, China

[2]Department of Pathology, The Second Affiliated Hospital of Hainan Medical University, Haikou 570311, Hainan Province, China

[#]Co-first authors, contributed equally to this work.

[*]Correspondence: Mingfa Wang, wwwmingfa@163.com; Wenjun Tang, twjlucky@163.com; The Second Affiliated Hospital of Hainan Medical University, No.368 Yehai Avenue, Longhua District, Haikou 570311, Hainan, China

ORCiD of authors: Wenjun Tang, https://orcid.org/0009-0001-3709-7219

Supplementary Figures

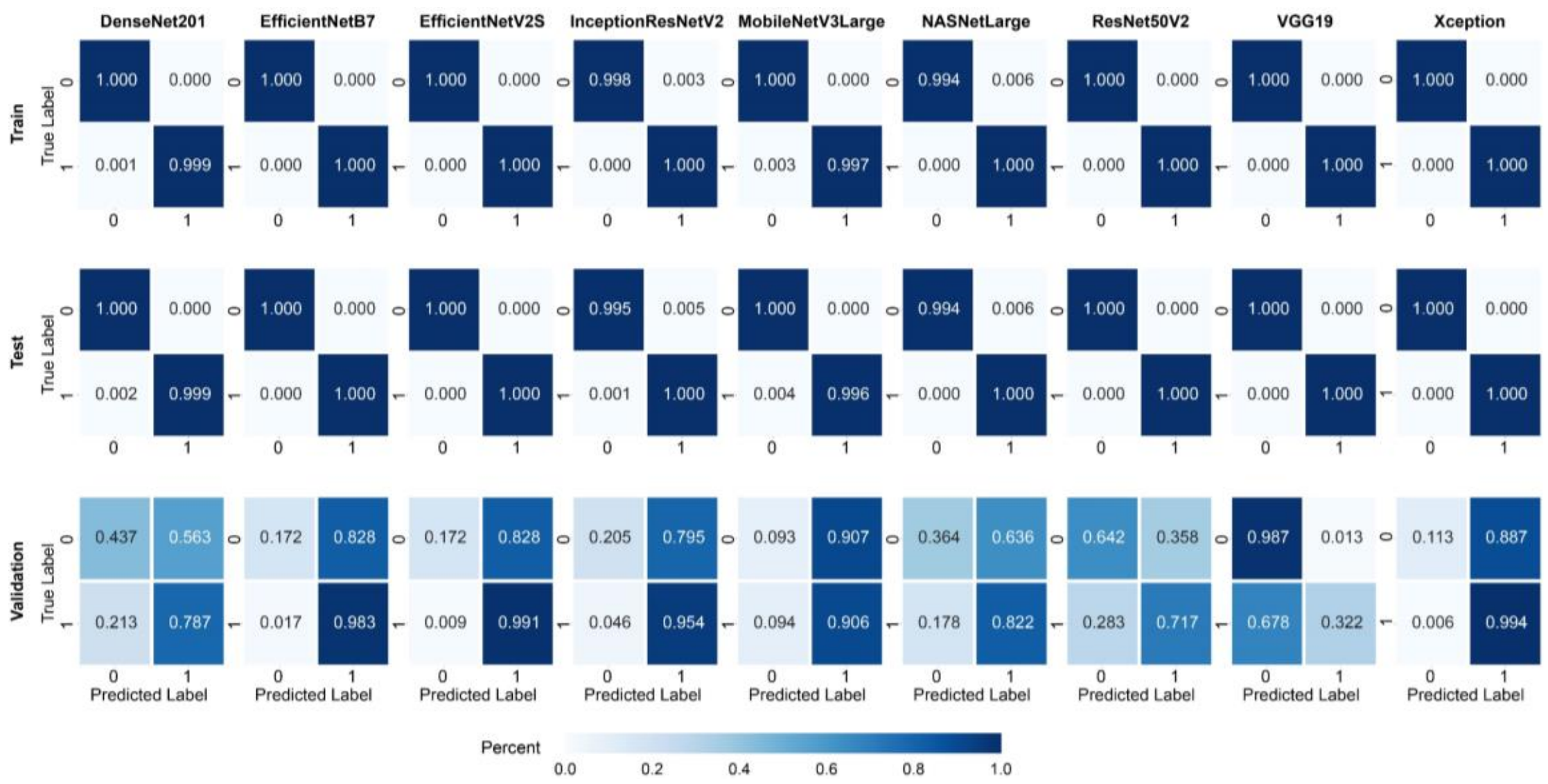

Supplementary Figure 1. Confusion matrix of single models

The 0 in the label stands for a normal lung sample, and 1 stands for a lung cancer sample.

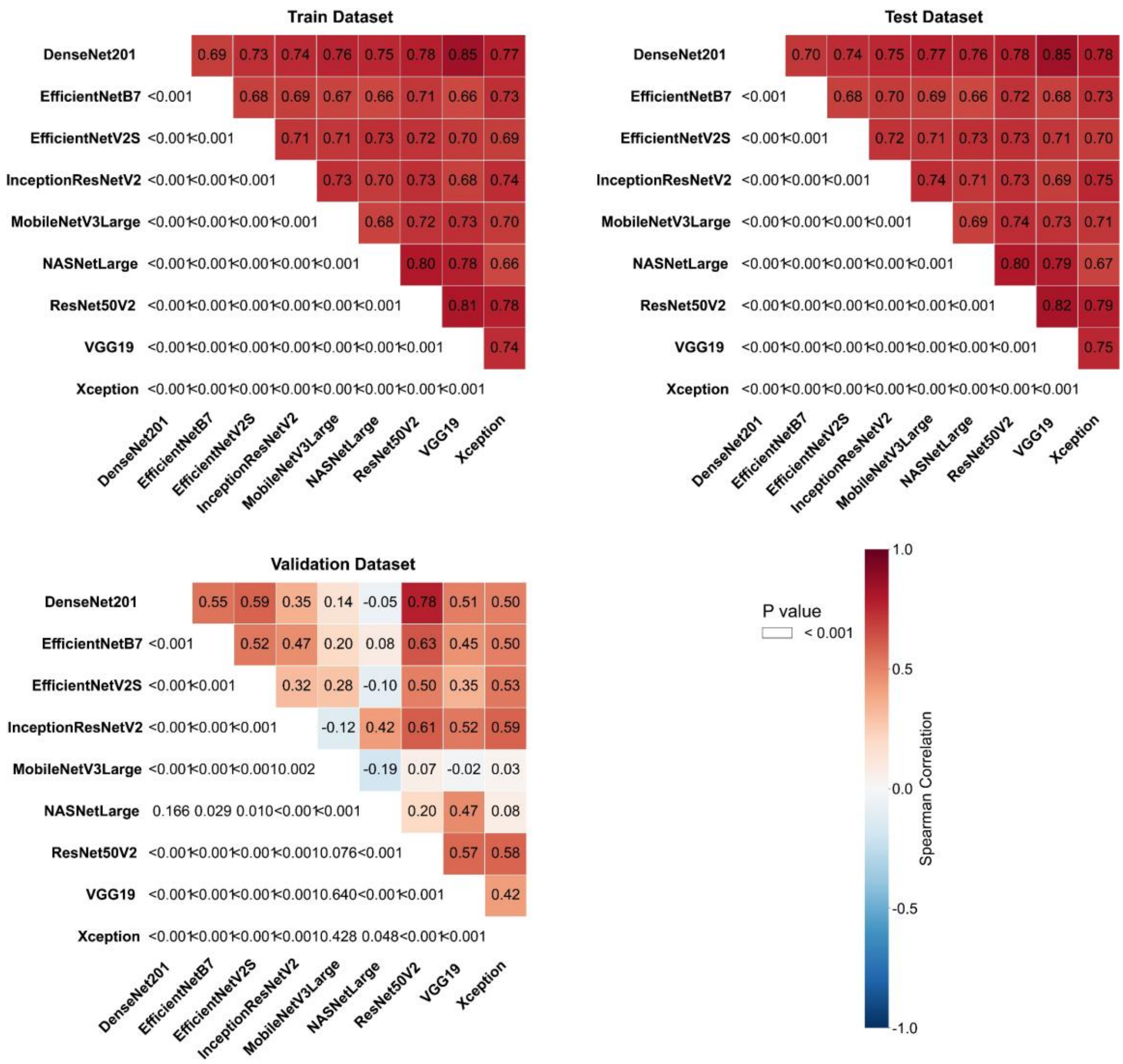

Supplementary Figure 2. Spearman correlation heatmap of single models

The upper triangle stands for the Spearman correlation coefficient, and the lower triangle stands for the corresponding P value.

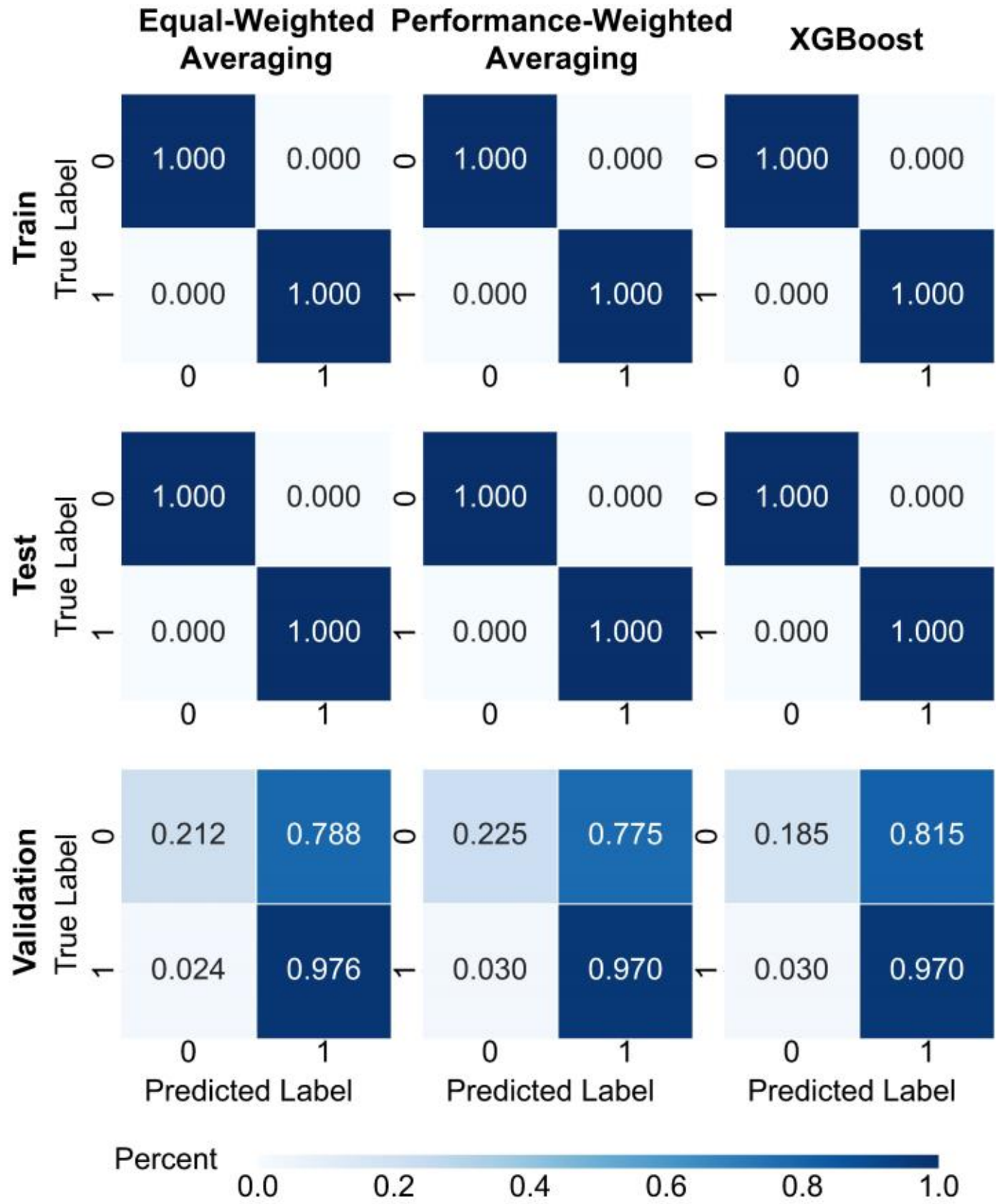

Supplementary Figure 3. Confusion matrix of ensemble models

The 0 in the label stands for a normal lung sample, and 1 stands for a lung cancer sample.

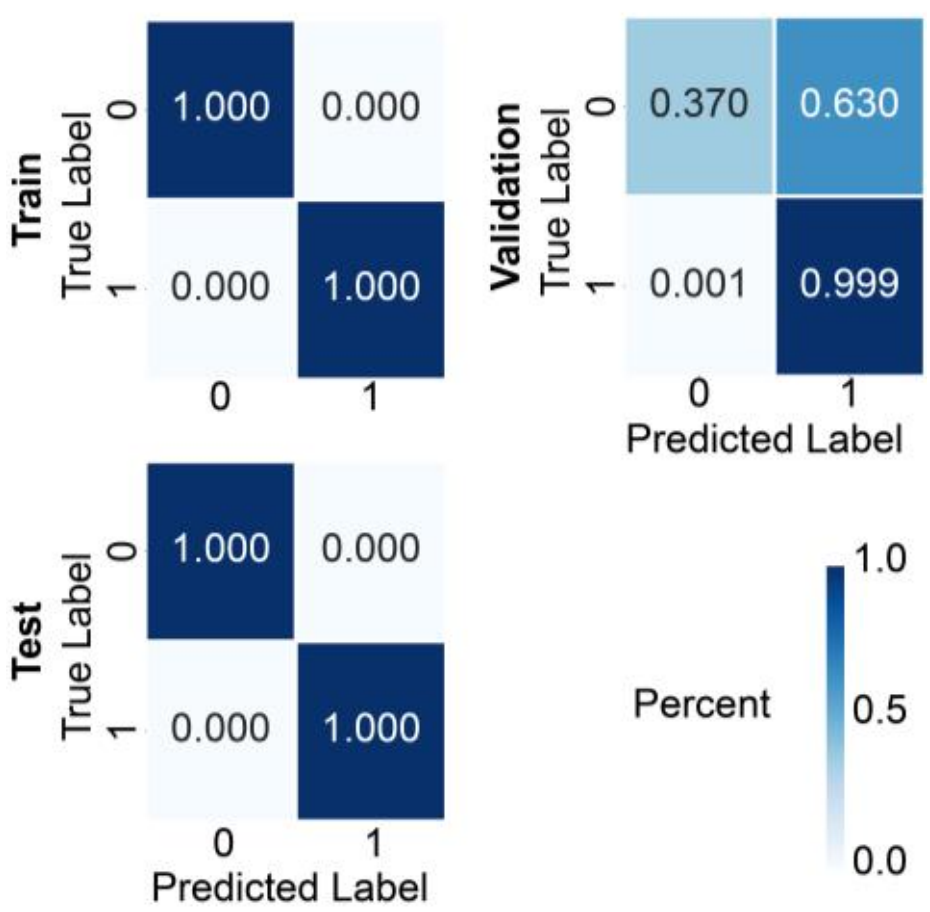

Supplementary Figure 4. Confusion matrix of optimized EfficientNetB7 model

The 0 in the label stands for a normal lung sample, and 1 stands for a lung cancer sample.
